# Diabetes mellitus burden among people living with HIV from the Asia‐Pacific region

**DOI:** 10.1002/jia2.25236

**Published:** 2019-01-29

**Authors:** Win M Han, Awachana Jiamsakul, Sasisopin Kiertiburanakul, Oon T Ng, Benedict LH Sim, Ly P Sun, Kinh Van Nguyen, Jun Y Choi, Man P Lee, Wing W Wong, Adeeba Kamarulzaman, Nagalingeswaran Kumarasamy, Fujie Zhang, Junko Tanuma, Cuong D Do, Romanee Chaiwarith, Tuti P Merati, Evy Yunihastuti, Sanjay Pujari, Rossana Ditangco, Suwimon Khusuwan, Jeremy Ross, Anchalee Avihingsanon, IeDEA Asia‐Pacific

**Affiliations:** ^1^ HIV‐NAT/Thai Red Cross AIDS Research Centre Bangkok Thailand; ^2^ The Kirby Institute UNSW Sydney Australia; ^3^ Faculty of Medicine Ramathibodi Hospital Mahidol University Bangkok Thailand; ^4^ Tan Tock Seng Hospital Singapore Singapore; ^5^ Hospital Sungai Buloh Sungai Buloh Malaysia; ^6^ National Center for HIV/AIDS, Dermatology & STDs Phnom Penh Cambodia; ^7^ National Hospital for Tropical Diseases Hanoi Vietnam; ^8^ Division of Infectious Diseases Department of Internal Medicine Yonsei University College of Medicine Seoul South Korea; ^9^ Queen Elizabeth Hospital Hong Kong SAR; ^10^ Taipei Veterans General Hospital Taipei Taiwan; ^11^ University Malaya Medical Centre Kuala Lumpur Malaysia; ^12^ Chennai Antiviral Research and Treatment Clinical Research Site (CART CRS) YRGCARE Medical Centre VHS Chennai India; ^13^ Beijing Ditan Hospital Capital Medical University Beijing China; ^14^ National Center for Global Health and Medicine Tokyo Japan; ^15^ Bach Mai Hospital Hanoi Vietnam; ^16^ Research Institute for Health Sciences Chiang Mai Thailand; ^17^ Faculty of Medicine Udayana University & Sanglah Hospital Bali Indonesia; ^18^ Faculty of Medicine Universitas Indonesia – Dr. Cipto Mangunkusumo General Hospital Jakarta Indonesia; ^19^ Institute of Infectious Diseases Pune India; ^20^ Research Institute for Tropical Medicine Muntinlupa City Philippines; ^21^ Chiangrai Prachanukroh Hospital Chiang Rai Thailand; ^22^ TREAT Asia, amfAR – The Foundation for AIDS Research Bangkok Thailand; ^23^ Tuberculosis Research Unit Faculty of Medicine Chulalongkorn University Bangkok Thailand

**Keywords:** diabetes mellitus, virologically suppressed PLHIV, non‐communicable diseases, antiretroviral therapy, comorbidities, Asia‐Pacific

## Abstract

**Introduction:**

Comorbidities including diabetes mellitus (DM) among people living with HIV (PLHIV) are of increasing clinical concerns in combination antiretroviral therapy (cART) era. We aimed to determine the incidence and risk factors of new‐onset DM among PLHIV in Asian settings.

**Methods:**

PLHIV from a regional observational cohort without DM prior to antiretroviral therapy (ART) initiation were included in the analysis. DM was defined as having a fasting blood glucose ≥126 mg/dL, glycated haemoglobin ≥6.5%, a two‐hour plasma glucose ≥200 mg/dL, or a random plasma glucose ≥200 mg/dL. A Cox regression model, stratified by site, was used to identify risk factors associated with DM.

**Results and discussion:**

Of the 1927 participants included, 127 were diagnosed with DM after ART initiation. Median follow‐up time from ART initiation to DM diagnosis was 5.9 years (interquartile range (IQR): 2.8 to 8.9 years). The crude incidence rate of DM was 1.08 per 100 person‐years (100 PYS), 95% confidence interval (CI) (0.9 to 1.3). In the multivariate analysis, later years of follow‐up (2011 to 2013: HR = 2.34, 95% CI 1.14 to 4.79, *p* = 0.02; and 2014 to 2017: HR = 7.20, 95% CI 3.27 to 15.87, *p* < 0.001) compared to <2010, older age (41 to 50 years: HR = 2.46, 95% CI 1.39 to 4.36, *p*  = 0.002; and >50 years: HR = 4.19, 95% CI 2.12 to 8.28, *p* < 0.001) compared to <30 years, body mass index (BMI) >30 kg/m^2^ (HR = 4.3, 95% CI 1.53 to 12.09, *p* = 0.006) compared to BMI <18.5 kg/m^2^, and high blood pressure (HR = 2.05, 95% CI 1.16 to 3.63, *p* = 0.013) compared to those without high blood pressure, were associated with developing DM. The hazard was reduced for females (HR = 0.47, 95% CI 0.28 to 0.80, *p* = 0.006).

**Conclusions:**

Type 2 DM in HIV‐infected Asians was associated with later years of follow‐up, high blood pressure, obesity and older age. This highlights the importance of monitoring and routine screening for non‐communicable diseases including DM as PLHIV age.

## 1 Introduction

People living with human immunodeficiency virus (PLHIV) have better prognosis and greater longevity because of the benefits of highly effective combination antiretroviral therapy (cART), more effective management strategies and improvements in patient monitoring 1, 2, 3, 4, 5. With increasing survival, non‐AIDS complications and comorbidities are important key factors influencing morbidity and mortality among PLHIV. Studies have pointed out metabolic disorders such as diabetes mellitus (DM) were common in PLHIV 6, 7. A study using nationally representative survey data from the U.S. showed that DM prevalence was 3.8% higher in HIV‐infected individuals compared with the uninfected general population 6. A report from the Data Collection on Adverse Events of Anti‐HIV Drugs (D:A:D) study showed that the incidence of DM was 5.7 per 1000 person‐years of follow‐up 8.

Use of certain protease inhibitors (PI)‐based regimen has been reported 9 to be associated with higher incidence of DM in the early antiretroviral therapy (ART) era, but the associations were less common with the newer classes of PI. Antiretrovirals (ARV) containing older classes of nucleoside reverse transcriptase inhibitors (NRTIs) such as stavudine or didanosine might also increase the risk of developing DM, probably due to insulin resistance caused by mitochondrial toxicities 10, 11, 12. Moreover, DM is commonly associated with other comorbidities such as hypertension and dyslipidaemia, which can result in increased risk of developing cardiovascular diseases 10, 13.

Non‐communicable diseases including DM have been increased dramatically over the past few decades in Asia 14, of which more than half of the global DM population are located in this region 15. However, DM prevalence data among PLHIV in Asia‐Pacific region is still sparse. The incidence of DM varied among HIV population (0.5 to 1.31 cases per 100 persons‐years of follow‐up) in HIV population 8, 10, 16. The incidence of DM in Asia varied from Western countries and the risk factors for the development of DM among PLHIV are understudied in the region. Hence, we assessed the incidence and risk factors of new‐onset DM among PLHIV after cART initiation in a regional observational cohort in the Asia‐Pacific region.

## 2 Methods

### 2.1 Study design and participants

This study was a longitudinal analysis exploring the incidence of new‐onset DM after cART initiation. The study participants were PLHIV enrolled in the TREAT Asia HIV Observational Database (TAHOD) between 2003 and 2017. The cohort and its methods have previously been characterized 17, 18, 19. The TAHOD is a collaborative observational cohort study that involves 20 sites in the Asia and Pacific region. The participating countries are Cambodia, China and Hong Kong SAR, India, Indonesia, Japan, Malaysia, the Philippines, Singapore, South Korea, Taiwan, Thailand and Vietnam. The recruitment began in 2003. As of March 2017, there were 9160 participants enrolled. Data transfer occurs every six months in March and September. TAHOD does not mandate regular visit schedule and all tests/interventions are performed according to the site's local practices. Participants were included in this analysis if they have been on cART for more than six months, did not have evidence of DM prior to start of cART, and had at least one of the following measurements after cART initiation: fasting blood glucose (FBG), glycated haemoglobin (HbA1C), two‐hour plasma glucose after 75 g oral glucose tolerance test (OGTT), or a random plasma glucose (RPG). Participants without DM screening prior to cART initiation were excluded from the study. Participant consent was deferred according to the individual participating sites and their institutional review boards, and is not required for all participants.

### 2.2 Outcomes

DM was defined as having a single measurement showing FBG ≥126 mg/dL, HbA1C ≥6.5%, a two‐hour plasma glucose level after OGTT ≥200 mg/dL, or a RPG ≥200 mg/dL, modified from the standard criteria for DM diagnosis from American Diabetes Association 20. However, we did not include data of hyperglycaemic symptoms for RPG ≥200 mg/dL. Also, we did not use secondary confirmation testing of FBG as our median FBG testing frequency was once per patient per year (interquartile range (IQR) 1 to 2).

### 2.3 Covariates

Time‐fixed covariates included age, sex, mode of HIV exposure, initial cART regimen, any exposure to stavudine or didanosine in their first‐line ART regimen, hepatitis B and C co‐infection, prior AIDS diagnosis, smoking and alcohol status. Time‐updated covariates included calendar year of follow‐up, viral load, CD4, body mass index (BMI), high blood pressure and dyslipidaemia. Dyslipidaemia was defined as a single laboratory result of a fasting cholesterol >200 mg/dL or triglycerides >150 mg/dL. High blood pressure was defined as having at least one measurement of systolic blood pressure >140 mmHg or diastolic blood pressure >90 mmHg. All variables were categorical in the regression analysis.

### 2.4 Statistical analysis

Factors associated with DM diagnosis after cART initiation were analysed using a Cox regression model stratified by site, to account for clustering within each site. Risk time for DM started from cART initiation and ended on date of first DM diagnosis, among participants who have been on cART for at least six months. Participants without DM events were censored on the date of last measurement for DM markers. Since the study was an intention‐to‐treat analysis, we included cART regimen and individual ART drugs as time‐fixed covariates. Pre‐ART VL and CD4 cell count were defined as measurements taken within six months prior to start of cART. Prior AIDS diagnosis was defined as having a CDC disease stage C prior to cART initiation. All variables measured were entirely observational according to site's local practices. Covariates from the univariate analysis with *p* < 0.10 were fitted in the multivariate model using backward stepwise selection process. Covariates with *p* < 0.05 in the multivariate model were considered significant.

Ethics approval were obtained from respective local ethics committees of all TAHOD‐participating sites, the Kirby Institute (data management and statistical analysis centre), and TREAT Asia/amfAR (coordinating centre). All data management and statistical analyses were performed using SAS software version 9.4 (SAS Institute Inc., Cary, NC, USA) and Stata software version 14.2 (Stata Corp., College Station, TX, USA).

## 3 Results and discussion

A total of 1927 PLHIV receiving cART with no evidence of prior DM were included from 20 sites from the participating countries in TAHOD cohort. Of participants with glucose parameters available prior to cART initiation, there were 228 participants with previous diagnosis of DM who were excluded from the study. Sites contributed a median of 61 participants (IQR 20 to 163 participants) in the analysis. The median age was 35 years (IQR 30 to 41) and the median CD4 cell count at ART initiation was 162 cells/μL (IQR: 59 to 248) (Table 1). The majority were males (74%) and acquired HIV via heterosexual route (57%). Hepatitis C virus co‐infection occurred in 11% of total PLHIV. 1497 (78%) and 364 (19%) of the participants were on NRTIs plus NNRTI (non‐nucleoside reverse transcriptase inhibitor) and NRTIs plus PI as initial cART regimen respectively.

**Table 1:** Patient characteristics

	Total patients (%)	Total DM
	N = 1927 (100)	N = 127 (7)
Median age at ART initiation (years)	Median = 35, IQR (30 to 41)	Median = 38, IQR (32 to 45)
Sex		
Male	1426 (74)	109 (86)
Female	501 (26)	18 (14)
HIV mode of exposure		
Heterosexual contact	1102 (57)	70 (55)
MSM	554 (29)	34 (27)
IDU	105 (5)	12 (9)
Other/Unknown	166 (9)	11 (9)
Pre‐ART Viral Load (copies/mL)	Median = 78,340, IQR (18,752 to 240,000)	Median = 72,865, IQR (16,397 to 490,000)
Pre‐ART CD4 (cells/μL)	Median = 162, IQR (59 to 248)	Median = 125, IQR (42 to 201)
Initial cART regimen		
NRTI + NNRTI	1497 (78)	102 (80)
NRTI + PI	364 (19)	22 (17)
Other combination	66 (3)	3 (2)
Stavudine in first‐line ART		
No	1382 (72)	80 (63)
Yes	545 (28)	47 (37)
Didanosine in first‐line ART		
No	1879 (98)	120 (94)
Yes	48 (2)	7 (6)
Hepatitis B co‐infection		
Negative	1599 (83)	100 (79)
Positive	142 (7)	16 (13)
Not tested	186 (10)	11 (9)
Hepatitis C co‐infection		
Negative	1422 (74)	96 (76)
Positive	204 (11)	13 (10)
Not tested	301 (16)	18 (14)
Prior AIDS diagnosis		
No	1367 (71)	79 (62)
Yes	560 (29)	48 (38)
Ever smoked		
No	755 (39)	41 (32)
Yes	663 (34)	48 (38)
Not reported	509 (26)	38 (30)
Ever above moderate or low risk drinking		
No	324 (17)	20 (16)
Yes	107 (6)	10 (8)
Not reported	1496 (78)	97 (76)
Pre‐ART BMI (kg/m^2^)	Median = 21, IQR (19 to 23)	Median = 22, IQR (19 to 25)
Pre‐ART systolic blood pressure (mmHg)	Median = 112, IQR (100 to 123)	Median = 110, IQR (100 to 120)
Pre‐ART diastolic blood pressure (mmHg)	Median = 70, IQR (63 to 80)	Median = 70, IQR (60 to 80)
Pre‐ART ALT (U/L)	Median = 29, IQR (19 to 45)	Median = 34, IQR (21 to 54)
Pre‐ART fasting blood glucose (mmol/L)	Median = 4.9, IQR (4.5 to 5.4)	Median = 5.4, IQR (4.7 to 6.1)
Pre‐ART random blood glucose (mmol/L)	Median = 5.2, IQR (4.9 to 5.4)	N/A

ART, antiretroviral therapy; BMI, body mass index; IDU, injecting drug users; IQR, interquartile range; MSM, men who have sex with men; NNRTI, non‐nucleoside reverse transcriptase inhibitor; NRTI, nucleoside reverse transcriptase inhibitors; PI, protease inhibitor.

There were 127 PLHIV (7%) who had DM after cART with an incidence rate of 1.08 per 100 person‐years (/100PYS) under a median follow‐up time of 5.9 years (IQR: 2.8 to 8.9 years). Of the 127 participants, 117 met the FBG criteria, 9 met the HbA1C criteria and 1 met the OGTT criteria for DM. The incidence rate for DM for those with HCV co‐infection was 1.18/100PYS which was higher than 1.05/100PYS for HCV negative participants, however the HR was not statistically significant when included in the univariate Cox regression analysis (*p* = 0.219). Factors associated with development of DM after ART initiation are shown in Table 2. Calendar year of follow‐up (*p* < 0.001), age (*p* < 0.001), sex (*p* = 0.004), BMI (*p* = 0.009), high blood pressure (*p* = 0.001) and dyslipidaemia (*p* = 0.059) were significant in the univariate analysis and included in the multivariate model.

**Table 2:** Factors associated with DM diagnosis after ART initiation

Time to DM stratified by site					Univariate		Multivariate	
	Number	Follow up (years)	No of DM	Incidence rate (/100PYS)	HR (95% CI)	*p*‐value	HR (95% CI)	*p*‐value
Total	1927	11,798	127	1.08				
Calendar year of follow‐up^a^								
≤2010		4936	19	0.38	1	<0.001	**1**	<0.001
2011 to 2013		3732	32	0.86	2.45 (1.21, 4.95)	0.013	**2.34 (1.14, 4.79)**	**0.020**
2014 to 2017		3131	76	2.43	7.76 (3.61, 16.66)	<0.001	**7.20 (3.27, 15.87)**	**<0.001**
Age at ART initiation (years)								
≤30	552	3246	22	0.68	1	<0.001	1	**<0.001**
31 to 40	847	5164	52	1.01	1.45 (0.87, 2.43)	0.157	1.28 (0.75, 2.17)	0.363
41 to 50	381	2482	37	1.49	2.83 (1.62, 4.94)	<0.001	**2.46 (1.39, 4.36)**	**0.002**
>50	147	906	16	1.77	4.37 (2.26, 8.46)	<0.001	**4.19 (2.12, 8.28)**	**<0.001**
Sex								
Male	1426	8949	109	1.22	1		1	
Female	501	2849	18	0.63	0.47 (0.28, 0.78)	0.004	**0.47 (0.28, 0.80)**	**0.006**
HIV mode of exposure								
Heterosexual contact	1102	6720	70	1.04	1	0.391		
MSM	554	3449	34	0.99	1.48 (0.80, 2.73)	0.207		
IDU	105	477	12	2.51	1.67 (0.78, 3.57)	0.184		
Other/Unknown	166	1152	11	0.96	1.33 (0.64, 2.76)	0.449		
HIV viral load (copies/mL)^a^								
≤1000		9326	94	1.01	1			
>1000		1000	12	1.20	1.56 (0.77, 3.16)	0.216		
Not done		1472	21	1.43				
CD4 cell count (cells/μL)^a^								
≤200		1732	23	1.33	1	0.615		
201 to 350		2717	22	0.81	0.86 (0.44, 1.68)	0.651		
351 to 500		3014	26	0.86	0.70 (0.35, 1.42)	0.322		
>500		4300	56	1.3	0.81 (0.42, 1.57)	0.529		
Not done		35	0	0				
Initial cART regimen								
NRTI + NNRTI	1497	8403	102	1.21	1	0.145		
NRTI + PI	364	3066	22	0.72	0.55 (0.27, 1.11)	0.095		
Other combination	66	329	3	0.91	1.54 (0.45, 5.23)	0.487		
Stavudine in first‐line ART?								
No	1382	8682	80	0.92	1			
Yes	545	3116	47	1.51	0.98 (0.61, 1.57)	0.924		
Didanosine in first‐line ART?								
No	1879	11,391	120	1.05	1			
Yes	48	406	7	1.72	1.85 (0.79, 4.37)	0.158		
Hepatitis B co‐infection								
Negative	1599	9791	100	1.02	1			
Positive	142	875	16	1.83	1.58 (0.91, 2.73)	0.102		
Not tested	186	1132	11	0.97				
Hepatitis C co‐infection								
Negative	1422	9131	96	1.05	1			
Positive	204	1106	13	1.18	0.65 (0.33, 1.29)	0.219		
Not tested	301	1561	18	1.15				
Prior AIDS diagnosis								
No	1367	8311	79	0.95	1			
Yes	560	3486	48	1.38	1.35 (0.91, 1.99)	0.137		
BMI (kg/m^2^)^a^								
<18.5		884	7	0.79	1	0.009	1	**0.025**
18.5 to 25.0		6816	68	1.00	1.32 (0.59, 2.95)	0.491	1.06 (0.47, 2.38)	0.892
25.0 to 30.0		1666	19	1.14	1.89 (0.76, 4.70)	0.168	1.17 (0.46, 2.97)	0.736
>30.0		333	11	3.30	5.39 (1.94, 14.97)	0.001	**4.30 (1.53, 12.09)**	**0.006**
Not reported		2098	22	1.05				
High blood pressure^a^								
No		7562	73	0.97	1		1	
Yes		1211	24	1.98	2.64 (1.52, 4.59)	0.001	**2.05 (1.16, 3.63)**	**0.013**
Not done		3025	30	0.99				
Dyslipidaemia^a^								
No		4703	34	0.72	1			
Yes		6371	81	1.27	1.49 (0.99, 2.24)	0.059		
Not reported		724	12	1.66				
Ever smoked								
No	755	4674	41	0.88	1			
Yes	663	4564	48	1.05	1.29 (0.83, 2.00)	0.263		
Not reported	509	2560	38	1.48				
Ever above moderate or low risk drinking								
No	324	2256	20	0.89	1			
Yes	107	694	10	1.44	1.15 (0.49, 2.73)	0.743		
Not reported	1496	8847	97	1.10				

*p*‐vales in bold represent significant covariates in the final model. Global *p*‐values are test for heterogeneity excluding missing values. Dyslipidaemia was defined as a single laboratory result of a fasting cholesterol >200 mg/dL or triglycerides >150 mg/dL. High blood pressure was defined as having at least one measurement of systolic blood pressure >140 mmHg or diastolic blood pressure >90 mmHg. ART, antiretroviral therapy; cART, combination antiretroviral therapy; IDU, injecting drug users; MSM, men who have sex with men; NNRTI, non‐nucleoside reverse transcriptase inhibitor; NRTI, nucleoside reverse transcriptase inhibitors; PI, protease inhibitor. ^a^Calendar year of follow‐up, CD4, VL, BMI, High blood pressure, dyslipidaemia are time‐updated variables.

In the multivariate analysis, factors associated with DM diagnosis included later years of follow‐up (years 2011 to 2013: HR = 2.34, 95% confidence interval (CI) 1.14 to 4.79, *p* = 0.02; and years 2014 to 2017: HR = 7.20, 95% CI 3.27 to 15.87, *p* < 0.001) compared to follow‐up years before 2010; older age at cART initiation (41 to 50 years: HR = 2.46, 95% CI 1.39 to 4.36, *p* = 0.002; and >50 years: HR = 4.19, 95% CI 2.12 to 8.28, *p* < 0.001) compared to age <30 years; having BMI >30 kg/m^2^ (HR = 4.3, 95% CI 1.53 to 12.09, *p* = 0.006) compared to BMI <18.5 kg/m^2^; and having high blood pressure (HR = 2.05, 95% CI 1.16 to 3.63, *p* = 0.013) compared to those without high blood pressure. The female sex was associated with 53% reduction in hazard for DM (HR = 0.47, 95% CI 0.28 to 0.80, *p* = 0.006) compared to males.

Overall incidence of new‐onset DM is 1.08 per 100PYS in a median follow‐up time of nearly six years after cART initiation among PLHIV from a cohort in the Asia‐Pacific region. Risk factors associated with new‐onset DM in this cohort includes older age, higher BMI and high blood pressure. The DM incidence is similar to previous studies 10, 16 in the Asia‐Pacific region, with a total of 11,798 person‐years of follow‐up among 1927 PLHIV in our multicenter cohort in the Asia‐Pacific, which includes 20 sites from 12 different countries and territories. The incidence rate of DM was higher among males (1.22/100PYS) compared to females (0.63/100PYS). Traditional risks factors such as age, BMI and high blood pressure were found to be predictors of DM, reflecting the emergence of non‐communicable disease comorbidities. In addition, we also found that the later years of follow‐up was associated with higher DM incidence, compared to follow‐up years before 2010. This could be partly due to the collection of OGTT, HbA1C and RPG results into the cohort after 2015, although the routine FBG median FBG testing frequency was the same as once per patient per year. We did not observe an association between DM and the use of baseline ARVs such as stavudine or didanosine in the initial regimen or the use of PIs‐ or NNRTIs‐based regimen as baseline ART.

Incident DM was more common in males, participants with increasing age and higher BMI, which are consistent with the risk factors among the general population. Moreover, there was no significant difference between HIV exposure risks (heterosexual, men who have sex with men (MSM) and injectable drug users) and incident DM in our study. DM incidence among PLHIV has varied by geographic region and country income level. The US Multicenter AIDS Cohort Study (MACS) 21 and Women's Interagency HIV Study (WIHS) 22, where the majority of the participants were African‐American and Hispanic/Latino, have reported higher incidence rates of DM, with 4.7/100PYS and 3.4/100PYS among HIV‐infected participants on ART in MACS and WIHS respectively. Our findings were similar to the incidence rates from the previous studies 10, 16, 23. However, lower rates have been reported from other cohorts such as Swiss HIV Cohort 24 and D:A:D study 8, with incidence rates of 0.44/100PYS and 0.57/100PYS respectively. Differences in DM incidence rates among the cohorts are possibly due to different ethnic backgrounds since Asians are more prone to have more visceral adipose tissue accumulation than European population, which could lead to higher chances of insulin resistance 25, 26. Additionally, the differences in DM incidence among cohorts may also be contributed by the differences in the length of follow‐up time and the definition of DM we used in the study which did not require a confirmation test of fasting blood sugar which can result in having lower specificity for detecting DM.

With regard to HIV‐ and ART‐related factors, we did not observe associations between DM and time‐updated CD4 cell counts, HIV viral load or prior AIDS‐defining events. Previous reports have suggested that persistent inflammation due to chronic infection may have an impact on the pathogenesis of DM 27. In addition to the effect of chronic inflammation of HIV infection on insulin resistance, past studies have shown an association of ART with DM development. Most of our participants (80%) who developed DM were on NNRTIs‐based regimen as initial cART. In this analysis, we did not find the link of either stavudine or didanosine with incident DM, even though the use of stavudine as initial cART regimen was high (28%) in our cohort. It is interesting to note that stavudine was suggested to have been associated with lipodystrophy 28 but not DM in the same population. Furthermore, certain PIs may contribute to the inhibition of glucose transporter (glucose transporter type 4 isoform, GLUT4) and the reduction in insulin sensitivity, which could lead to decreased glucose uptake in peripheral adipose tissues and consequently result in the development of insulin resistance 29. Contrary to the previous studies 9, 24, we found no association between DM and PI‐ versus NNRTI‐based initial cART regimen.

It is noteworthy that high blood pressure was also associated with DM incidence in our cohort. This is consistent with the findings from the general population without HIV infection 30. HIV‐infected participants with high blood pressure and DM may also have risks for multiple comorbidities and polypharmacy. This will increase the pill burden and have a high possibility to have drug‐to‐drug interactions. Therefore, proper treatment and risk reduction strategies such as diet and exercise should be implemented in those who have higher BMI and abnormal blood pressure. Our findings showed that traditional risk factors were associated with DM development among Asian HIV‐infected individuals, suggesting the needs for the importance of clinicians to timely diagnose and properly manage DM in HIV‐infected individuals. Even though we did not observe the use of ARVs to be an additional risk for the occurrence of DM, however it remains crucial to monitor and evaluate the potential toxicities from different ARVs used.

The limitations of the study include the unavailability of other indicators for central obesity such as abdominal fat and waist‐hip circumference ratio. We therefore were not able to evaluate the effects of these indicators on the development of DM in our analysis. Also, another limitation of this study is that we did not include HIV‐negative controls to compare the incidence rates of DM between the groups. Due to the limitations in DM testing data, such as FBG, we did not use a second confirmatory testing for DM, which could possibly lead to inaccurate estimation of our cohort's DM incidence rate. As our cohort recruit participants based on the likelihood of remaining in care, the study population may not represent patients typically seen at the clinical sites. Finally, our study has limitations for inability to adjust all the unobserved confounding factors due to the observational nature of the cohort.

## 4 Conclusions

Our analysis shows DM in PLHIV is common in settings from Asian countries. Traditional risk factors such as age, sex, high blood pressure and BMI were found to be associated with the development of DM in our cohort. Careful assessment and routine screening for DM and other co‐existing comorbid conditions, especially among older and obese PLHIV, are essential.

## Competing interests

The authors do not have any competing interests to declare.

## Authors’ contributions

WH, AJ and AA contributed to the concept development. SK, OTN, BS, LPS, KVN, JYC, MPL, WWW, AK, NK, FZ, JT, CDD, RC, TPM, EY, SP, RD, SK and AA contributed data for the analysis. AJ performed the statistical analysis. WH wrote the first draft of the manuscript. All authors commented on the draft manuscript and approved of the final manuscript.

## Appendix 1

### 1.1

TAHOD study members: PS Ly* and V Khol, National Center for HIV/AIDS, Dermatology & STDs, Phnom Penh, Cambodia; FJ Zhang* †, HX Zhao and N Han, Beijing Ditan Hospital, Capital Medical University, Beijing, China; MP Lee*, PCK Li, W Lam and YT Chan, Queen Elizabeth Hospital, Hong Kong SAR; N Kumarasamy*, S Saghayam and C Ezhilarasi, Chennai Antiviral Research and Treatment Clinical Research Site (CART CRS), YRGCARE Medical Centre, VHS, Chennai, India; S Pujari*, K Joshi, S Gaikwad and A Chitalikar, Institute of Infectious Diseases, Pune, India; S Sangle*, V Mave and I Marbaniang, BJ Government Medical College and Sassoon General Hospital, Pune, India; TP Merati*, DN Wirawan and F Yuliana, Faculty of Medicine Udayana University & Sanglah Hospital, Bali, Indonesia; E Yunihastuti*, D Imran and A Widhani, Faculty of Medicine Universitas Indonesia ‐ Dr. Cipto Mangunkusumo General Hospital, Jakarta, Indonesia; J Tanuma*, S Oka and T Nishijima, National Center for Global Health and Medicine, Tokyo, Japan; JY Choi*, Na S and JM Kim, Division of Infectious Diseases, Department of Internal Medicine, Yonsei University College of Medicine, Seoul, South Korea; BLH Sim*, YM Gani, and NB Rudi, Hospital Sungai Buloh, Sungai Buloh, Malaysia; A Kamarulzaman*, SF Syed Omar, S Ponnampalavanar and I Azwa, University Malaya Medical Centre, Kuala Lumpur, Malaysia; R Ditangco*, MK Pasayan and ML Mationg, Research Institute for Tropical Medicine, Muntinlupa City, Philippines; WW Wong*, WW Ku and PC Wu, Taipei Veterans General Hospital, Taipei, Taiwan; OT Ng* ‡, PL Lim, LS Lee and Z Ferdous, Tan Tock Seng Hospital, Singapore; A Avihingsanon*, S Gatechompol, P Phanuphak and C Phadungphon, HIV‐NAT/Thai Red Cross AIDS Research Centre, Bangkok, Thailand; S Kiertiburanakul*, A Phuphuakrat, L Chumla and N Sanmeema, Faculty of Medicine Ramathibodi Hospital, Mahidol University, Bangkok, Thailand; R Chaiwarith*, T Sirisanthana, W Kotarathititum and J Praparattanapan, Research Institute for Health Sciences, Chiang Mai, Thailand; S Khusuwan*, P Kantipong and P Kambua, Chiangrai Prachanukroh Hospital, Chiang Rai, Thailand; KV Nguyen*, HV Bui, DTH Nguyen and DT Nguyen, National Hospital for Tropical Diseases, Hanoi, Vietnam; CD Do*, AV Ngo and LT Nguyen, Bach Mai Hospital, Hanoi, Vietnam; AH Sohn*, JL Ross* and B Petersen, TREAT Asia, amfAR ‐ The Foundation for AIDS Research, Bangkok, Thailand; MG Law*, A Jiamsakul* and D Rupasinghe, The Kirby Institute, UNSW Sydney, NSW, Australia. * TAHOD Steering Committee member; † Steering Committee Chair; ‡ co‐Chair.

## References

[1] Antiretroviral Therapy Cohort C . Life expectancy of individuals on combination antiretroviral therapy in high‐income countries: a collaborative analysis of 14 cohort studies. Lancet. 2008; 372(9635):293–9. 18657708 10.1016/S0140-6736(08)61113-7 PMC3130543

[2] De La Mata NL , Kumarasamy N , Khol V , Ng OT , Van Nguyen K , Merati TP , et al. Improved survival in HIV treatment programs in Asia. Antivir Ther. 2016;21(6):517–27. 26961354 10.3851/IMP3041 PMC4980281

[3] Boyd MA . Improvements in antiretroviral therapy outcomes over calendar time. Curr Opin HIV AIDS. 2009;4(3):194–9. 19532050 10.1097/COH.0b013e328329fc8d

[4] Deeks SG , Lewin SR , Havlir DV . The end of AIDS: HIV infection as a chronic disease. Lancet. 2013;382(9903):1525–33. 24152939 10.1016/S0140-6736(13)61809-7 PMC4058441

[5] Group ISS , Lundgren JD , Babiker AG , Gordin F , Emery S , Grund B , Sharma S , et al. Initiation of antiretroviral therapy in early asymptomatic HIV infection. N Engl J Med. 2015; 373(9):795–807. 26192873 10.1056/NEJMoa1506816 PMC4569751

[6] Hernandez‐Romieu AC , Garg S , Rosenberg ES , Thompson‐Paul AM , Skarbinski J . Is diabetes prevalence higher among HIV‐infected individuals compared with the general population? Evidence from MMP and NHANES 2009–2010. BMJ Open Diabetes Res Care. 2017;5(1):e000304. 10.1136/bmjdrc-2016-000304 PMC5293823 28191320

[7] Samaras K . Prevalence and pathogenesis of diabetes mellitus in HIV‐1 infection treated with combined antiretroviral therapy. J Acquir Immune Defic Syndr. 2009;50(5):499–505. 19223782 10.1097/QAI.0b013e31819c291b

[8] De Wit S , Sabin CA , Weber R , Worm SW , Reiss P , Cazanave C , et al. Incidence and risk factors for new‐onset diabetes in HIV‐infected patients: the Data Collection on Adverse Events of Anti‐HIV Drugs (D:A:D) study. Diabetes Care. 2008;31(6):1224–9. 18268071 10.2337/dc07-2013 PMC2746200

[9] Justman JE , Benning L , Danoff A , Minkoff H , Levine A , Greenblatt RM , et al. Protease inhibitor use and the incidence of diabetes mellitus in a large cohort of HIV‐infected women. J Acquir Immune Defic Syndr. 2003;32(3):298–302. 12626890 10.1097/00126334-200303010-00009

[10] Putcharoen O , Wattanachanya L , Sophonphan J , Siwamogsatham S , Sapsirisavat V , Gatechompol S , et al. New‐onset diabetes in HIV‐treated adults: predictors, long‐term renal and cardiovascular outcomes. AIDS. 2017;31(11):1535–43. 28398958 10.1097/QAD.0000000000001496

[11] Garcia‐Benayas T , Rendon AL , Rodriguez‐Novoa S , Barrios A , Maida I , Blanco F , et al. Higher risk of hyperglycemia in HIV‐infected patients treated with didanosine plus tenofovir. AIDS Res Hum Retroviruses. 2006;22(4):333–7. 16623636 10.1089/aid.2006.22.333

[12] Brambilla AM , Novati R , Calori G , Meneghini E , Vacchini D , Luzi L , et al. Stavudine or indinavir‐containing regimens are associated with an increased risk of diabetes mellitus in HIV‐infected individuals. AIDS. 2003;17(13):1993–5. 12960836 10.1097/00002030-200309050-00022

[13] Abebe SM , Getachew A , Fasika S , Bayisa M , Girma Demisse A , Mesfin N . Diabetes mellitus among HIV‐infected individuals in follow‐up care at University of Gondar Hospital, Northwest Ethiopia. BMJ Open. 2016;6(8):e011175. 10.1136/bmjopen-2016-011175 PMC5013550 27540099

[14] Yoon KH , Lee JH , Kim JW , Cho JH , Choi YH , Ko SH , et al. Epidemic obesity and type 2 diabetes in Asia. Lancet. 2006;368(9548):1681–8. 17098087 10.1016/S0140-6736(06)69703-1

[15] Ramachandran A , Snehalatha C , Shetty AS , Nanditha A . Trends in prevalence of diabetes in Asian countries. World J Diabetes. 2012;3(6):110–7. 22737281 10.4239/wjd.v3.i6.110 PMC3382707

[16] Lo YC , Chen MY , Sheng WH , Hsieh SM , Sun HY , Liu WC , et al. Risk factors for incident diabetes mellitus among HIV‐infected patients receiving combination antiretroviral therapy in Taiwan: a case‐control study. HIV Med. 2009;10(5):302–9. 19220492 10.1111/j.1468-1293.2008.00687.x

[17] Zhou J , Kumarasamy N , Ditangco R , Kamarulzaman A , Lee CK , Li PC , et al. The TREAT Asia HIV Observational Database: baseline and retrospective data. J Acquir Immune Defic Syndr. 2005;38(2):174–9. 15671802 10.1097/01.qai.0000145351.96815.d5 PMC3070962

[18] Jung IY , Boettiger D , Wong WW , Lee MP , Kiertiburanakul S , Chaiwarith R , et al. The treatment outcomes of antiretroviral substitutions in routine clinical settings in Asia; data from the TREAT Asia HIV Observational Database (TAHOD). J Int AIDS Soc. 2017; 20(4):e25016. 10.1002/jia2.25016 PMC5810317 29243388

[19] Ahn JY , Boettiger D , Law M , Kumarasamy N , Yunihastuti E , Chaiwarith R , et al. Implementation and operational research: effects of CD4 monitoring frequency on clinical end points in clinically stable HIV‐infected patients with viral suppression. J Acquir Immune Defic Syndr. 2015;69(3):e85–92. 25850606 10.1097/QAI.0000000000000634 PMC4506699

[20] Classification and Diagnosis of Diabetes . Standards of Medical Care in Diabetes – 2018. Diabetes Care. 2018;41 Suppl 1:S1–2. 29222373 10.2337/dc18-S002

[21] Brown TT , Cole SR , Li X , Kingsley LA , Palella FJ , Riddler SA , et al. Antiretroviral therapy and the prevalence and incidence of diabetes mellitus in the multicenter AIDS cohort study. Arch Intern Med. 2005;165(10):1179–84. 15911733 10.1001/archinte.165.10.1179

[22] Tien PC , Schneider MF , Cole SR , Levine AM , Cohen M , DeHovitz J , et al. Antiretroviral therapy exposure and incidence of diabetes mellitus in the Women's Interagency HIV Study. AIDS. 2007;21(13):1739–45. 17690572 10.1097/QAD.0b013e32827038d0

[23] McMahon CN , Petoumenos K , Hesse K , Carr A , Cooper DA , Samaras K . High rates of incident diabetes and prediabetes are evident in men with treated HIV followed for 11 years. AIDS. 2017;32(4):451–9. 10.1097/QAD.0000000000001709 29381559

[24] Ledergerber B , Furrer H , Rickenbach M , Lehmann R , Elzi L , Hirschel B , et al. Factors associated with the incidence of type 2 diabetes mellitus in HIV‐infected participants in the Swiss HIV Cohort Study. Clin Infect Dis. 2007;45(1):111–9. 17554711 10.1086/518619

[25] Ramachandran A , Ma RC , Snehalatha C . Diabetes in Asia. Lancet. 2010;375(9712):408–18. 19875164 10.1016/S0140-6736(09)60937-5

[26] Lear SA , Humphries KH , Kohli S , Chockalingam A , Frohlich JJ , Birmingham CL . Visceral adipose tissue accumulation differs according to ethnic background: results of the Multicultural Community Health Assessment Trial (M‐CHAT). Am J Clin Nutr. 2007;86(2):353–9. 17684205 10.1093/ajcn/86.2.353

[27] Capeau J , Bouteloup V , Katlama C , Bastard JP , Guiyedi V , Salmon‐Ceron D , et al. Ten‐year diabetes incidence in 1046 HIV‐infected patients started on a combination antiretroviral treatment. AIDS. 2012;26(3):303–14. 22089377 10.1097/QAD.0b013e32834e8776

[28] Han SH , Zhou J , Saghayam S , Vanar S , Phanuphak N , Chen YM , et al. Prevalence of and risk factors for lipodystrophy among HIV‐infected patients receiving combined antiretroviral treatment in the Asia‐Pacific region: results from the TREAT Asia HIV Observational Database (TAHOD). Endocr J. 2011;58(6):475–84. 21521929 10.1507/endocrj.k10e-407 PMC3329967

[29] Murata H , Hruz PW , Mueckler M . Indinavir inhibits the glucose transporter isoform Glut4 at physiologic concentrations. AIDS. 2002;16(6):859–63. 11919487 10.1097/00002030-200204120-00005

[30] Mengesha AY . Hypertension and related risk factors in type 2 diabetes mellitus (DM) patients in Gaborone City Council (GCC) clinics, Gaborone, Botswana. Afr Health Sci. 2007;7(4):244–5. 21499491 PMC3074377

